# LncRNA FOXD3-AS1 Promotes the Malignant Progression of Nasopharyngeal Carcinoma Through Enhancing the Transcription of YBX1 by H3K27Ac Modification

**DOI:** 10.3389/fonc.2021.715635

**Published:** 2021-07-29

**Authors:** Huiyun Yang, Yuliang Pan, Jun Zhang, Long Jin, Xi Zhang

**Affiliations:** ^1^ Department of Oncology, Xiangya Hospital, Central South University, Changsha, China; ^2^ Department of Oncology, Third Xiangya Hospital, Central South University, Changsha, China

**Keywords:** FOXD3-AS1, nasopharyngeal carcinoma, YBX1, H3K27ac, lnc RNA

## Abstract

**Background:**

Long noncoding RNAs (lncRNAs) can affect the progression of various tumors, including nasopharyngeal carcinoma (NPC). Here, lncRNA FOXD3-AS1 is highly expressed in NPC tissues through bioinformatics analysis and related to the malignant progression of NPC.

**Methods:**

Bioinformatics analysis and real-time reverse transcription quantitative PCR(RT-qPCR) assay were applied to identify the expression of FOXD3-AS1 in NPC tissues and cells. Specific short hairpin RNAs (shRNAs) or overexpression plasmids were used to knockdown or upregulate FOXD3-AS1 in NPC cells. The effect of FOXD3-AS1 on proliferation and metastasis of NPC was confirmed by CCK8, colony formation, transwell assays *in vitro* and mouse tumor growth and metastasis models *in vivo*, of which the mechanism was explored by RNA pull down, mass spectrometry (MS), RNA Immunoprecipitation (RIP), chromatin immunoprecipitation (CHIP) and luciferase assays.

**Results:**

FOXD3-AS1 was highly expressed in NPC tissues and cells. Knockdown of FOXD3-AS1 significantly inhibited proliferation, migration, and invasion of NPC cells *in vitro* and *vivo*. FOXD3-AS1 could specifically bind to YBX1 and have a positive effect on the expression of YBX1. Bioinformatics analysis showed that the promoter of YBX1 had a high enrichment of H3K27ac, which promote mRNA transcription and protein translation of YBX1. Moreover, overexpression of YBX1 could reverse the proliferation, migration and invasion arrest caused by FOXD3-AS1 knockdown.

**Conclusion:**

LncRNA FOXD3-AS1 is highly expressed and promotes malignant phenotype in NPC, which may provide a new molecular mechanism for NPC.

## Introduction

Nasopharyngeal carcinoma (NPC) is a prevalent cancer in South China and Southeast Asia and is often associated with the infection of Epstein-Barr virus (1). At present, the effective treatment of nasopharyngeal carcinoma is mainly radiotherapy or radiotherapy combined with adjuvant therapy (2). With the development of intense-modulated radiation therapy (3), the optimization of chemotherapy regimens (4) and the individualized implementation of chemotherapy (5), the prognosis of nasopharyngeal cancer has been greatly improved but some patients still end up with poor outcomes. The tumor, node, metastasis (TNM) staging system has prognostic significance for cancers, including NPC. The 5-year overall survival rate (OS) of locally advanced nasopharyngeal carcinoma is 68% ~ 80% and the main reason of treatment failure is distant metastasis (6). However, outcomes vary greatly even patients are at the same stage, because the current staging system does not take into account patients’ basic physical conditions, such as age and tumor size, which have been proved to be independent prognostic factors in other studies (7, 8). It seems a good idea to explain the differences of outcomes of patients at the molecular level. Many molecular markers have been identified to be associated with the prognosis of nasopharyngeal carcinoma, such as methylated modified genes (9), micro RNAs (10) and long non-coding RNAs (lncRNAs) (11), but the molecular mechanisms remain to be further studied. Therefore, there is an urgent need to find molecular targets and explore mechanisms to conduct individual therapy.

In recent years, lncRNAs have brought our attention (12, 13). Abnormal expression of lncRNAs matters much in the progression of the tumor. When the expression of lnc RNA SNHG12 is up-regulated, the ability of proliferation and metastasis of tumors is enhanced (14). Knockdown of lncRNA XIST inhibits malignant behavior of nasopharyngeal carcinoma by regulating miR-381-3p/NEK5 axis (15). By analyzing GSE datasets, we find that lncRNA FOXD3 is highly expressed in nasopharyngeal carcinoma. Chen X et al. have espoused that highly expressed FOXD3-AS1 targeting the miR-325/MAP3K2 axis can promote the development of cutaneous malignant melanoma (16). Chen Y et al. have proposed that the inhibition of FOXD3-AS1 can suppress progression of thyroid cancer by inhibiting TGF-β1/Smads signaling pathway (17). As for NPC, Hu J et al. have suggested that FOXD3-AS1 is up-regulated in NPC and aims at upregulating the FOXD3 expression by binding to miR-185-3p thus affects progression of tumor (18). However, mechanisms of FOXD3-AS1 effecting the malignant progression of nasopharyngeal carcinoma remain further exploration.

Therefore, based on the hints from the GSE datasets (GSE61218 and GSE126683), we verified that the expression of FOXD3-AS1 in NPC cell lines was consistent with the datasets by real-time reverse transcription quantitative PCR (RT-qPCR) assays, then the relationship between the expression level and the biological behavior of nasopharyngeal carcinoma was studied further. Combined with sequencing analysis, we identified and verified its downstream targets of FOXD3-AS1, thus explaining its relationship with the occurrence and development of nasopharyngeal carcinoma.

## Materials and Methods

### Bioinformation

To analyze the expression of lncRNA FOXD3-AS1 in NPC tissues, we used Gene Expression Omnibus (GEO) data sets (GSE61218 and GSE126683) to identify the expression of FOXD3-AS1 in NPC tissues through significant analysis of microarray (SAM). LNCipedia was used to predict the ability of encoding proteins (19). AnnoLnc was adopted to forecast the secondary structure of lncRNA FOXD3-AS1 (20). UCSC Genome Browser was used to predict the promoter region of YBX1.

### Cell Culture

All the cell lines were purchased from the American Type Culture Collection (ATCC, Manassas, VA, USA). Human NPC cell lines were cultured using Roswell Park Memorial Institute (RPMI)-1640 medium (Invitrogen, USA) added with 10% fetal bovine serum (FBS, Invitrogen, USA). Nasopharyngeal normal epithelium cell line NP69 was incubated in Keratinocyte serum-free medium (KSFM, Invitrogen, USA).

### Real-Time Reverse Transcription Quantitative PCR (RT-qPCR)

Total RNA was extracted using TRIzol reagent (Invitrogen, USA). Nuclear and cytoplasmic RNA was extracted and purified by the NE-PER Nuclear and Cytoplasmic Extraction Reagents (Invitrogen, USA) and following the manufacturer’s protocols. Then, reverse transcription of RNA into cDNA was carried out by M-MLV reverse transcriptase (Promega, USA). Real-time PCR assays were carried out by SYBR Green qPCR SuperMix-UDG reagents (Invitrogen, USA) with a CFX96 Touch sequence detection (Bio-Rad, USA). GAPDH was used as the endogenous control for genes mentioned in this article. First, results of the QPCR were normalized through the internal reference gene (GAPDH) and then calculated the relative quantification by the formula of 2^一△△CT^. All the final results came from three replicates. All primer sequences were provided in **Supplemental Table 1**.

### Vector Constructure, Cell Transfection and Lentiviral Infection

Two short hairpin RNAs (shRNAs) aimed to down-regulate the expression of FOXD3-AS1 were designed by BLOCK-iT™ RNAi Designer (ThermoFisher Scientific, USA). The nucleotide sequences of shRNA#1 and shRNA#2 were shown in **Supplemental Table 1**. The full-length of lncRNA FOXD3-AS1 was cloned from SUNE1 cell cDNA and cloned into the pcDNA3.1(+) or pcDNA3.1(-) to construct over expression plasmid. The wild-type and mutant-type promoters of YBX1 were cloned into the pGL3 vector (Addgene, USA). Knockdown or overexpression of FOXD3-AS1 was performed through transiently transfection using lipotamine 3000. The shFOXD3-AS1 or its scramble control (shCtrl) plasmid was co-transfected into HEK293T cells with the lentivirus packaging plasmids psPAX2 and pMD2G (Addgene, USA). The lentivirus supernatant was collected after 48h incubated and used to infect SUNE1 cells, and positive stably transfected cells were selected and maintained using puromycin.

### CCK-8 and Colony Formation Assays

HOEN1 and SUNE1 cells (1x10^3^) were incubated in 96-well plates for 5 days (0-4days). Then, we added ten microliters CCK-8 (Dojindo, Japan) per well to 96 well plates daily and incubator for two hours and the absorbance were measured at 450 nm. For the colony formation assays, 500 cells per well were planted in 6-well plates incubated with 2 ml medium. About 10 days of incubation, the cell colonies were fixed with methanol and stained with crystal violet to observe and count the number of colonies under a microscope.

### Migration and Invasion Assays

Transwell chambers (8 mm pores, Corning, USA) were used to performed migration and invasion assays and pre-coated with or without Matrigel (BD Biosciences, USA) for invasion or migration assays. First, we placed 200 ul of serum-free medium containing 5×10^4^ cells in each upper chamber and 500 ul of medium with 20% FBS in the lower chambers. After 14 hours (migration assays) or 21 hours (invasion assays), the cells in the upper chambers were fixed with formaldehyde, stained with crystal violet, observed, counted, and photographed with a microscope.

### Nucleo-Cytoplasmic Separation Experiments

NE-PER Nuclear and Cytoplasmic Extraction Reagents (ThermoFisher Scientific, USA) was utilized to isolate nuclear and cytoplasmic material. Firstly, 2*10^6^ suspended HONE1 or SUNE1 cells were centrifuged at 500g for 3 minutes, and the supernatant was discarded. Then, Cytoplasmic Extraction Reagent I (CER I), Cytoplasmic Extraction Reagent II (CER II) and Nuclear Extraction Reagent (NER) were added to obtain the nuclear and cytoplasmic extracts, respectively, and finally, trizol (Invitrogen, USA) was added to extract the nuclear and cytoplasmic RNAs. Reverse transcription and qPCR assays were performed to obtain the contents of FOXD3-AS1, GAPDH and U6 in the nucleus and cytoplasm of the cells, respectively.

### Fluorescence *In Situ* Hybridization (FISH) Assays

FOXD3-AS1 FISH probes (Fluorescent Probe-594) were generated from RiboBio. After the cells were planted overnight, cells were fixed with paraformaldehyde and incubated with probes according to the manufacturer’s instructions. Then, incubated with fluorescent secondary antibody Alexa Fluor^®^ 488 (Invitrogen, USA), finally incubated with 4’,6-diamidino-2-phenylindole (DAPI; Sigma, USA) for nuclei dyeing. The images were screened using fluorescence microscope (Olympus FV1000, Japan).

### RNA Pull Down Assays

FOXD3-AS1 sense and anti-sense plasmids were transcribed and biotin-labeled by the MEGAscript™ T7 Transcription Kit (Thermo Fisher Scientific, USA) and the Pierce™ RNA 3’ End Desthiobiotinylation Kit (Thermo Fisher Scientific, USA) *in vitro*. The pull-down assays were performed using the Magnetic RNA-Protein Pull-Down Kit (Thermo Fisher Scientific, USA). The proteins pulled down were subjected for Mass spectrometry (FitGene Biotechnology, China) and detected by western blotting.

### Western Blot Assays

Total protein of NPC cells was extracted using RIPA lysis buffer (Beyotime, China) and subjected to SDS-PAGE gel electrophoresis. The proteins were transferred into PVDF membrane (Merck Millipore, USA). The membrane was incubated with following primary antibody: anti-YBX1 (1:2000; Cell Signaling Technology, USA) or anti-GAPDH (1:5000; Cell Signaling Technology, USA). After incubated with secondary antibodies, the protein bands were defined by Chemiluminescence instrument (Bio-Rad, USA).

### RNA Immunoprecipitation (RIP) Assays

Cell lysates of NPC cells were incubated with Protein A/G Plus Agarose (Thermo Fisher Scientific, USA) at 4°C for 1h, and then incubated with 3.0 μg antibody of rabbit anti-YBX1 or normal control rabbit anti-IgG (Sigma-Aldrich, USA) at 4°C for 3h. The immunoprecipitated RNAs were purified and detected by RT-qPCR. Relative primers were listed in **Supplemental Table 1**.

### Chromatin Immunoprecipitation (ChIP) Assays

ChIP assays were conducted using anti-H3K27Ac by the Pierce™ Magnetic ChIP Kit (Thermo Fisher Scientific, USA) and following the manufacturers’ protocols. First, 5*10^6^ cells were cross-linked using formaldehyde, then the cross-linking was terminated using glycine and the lysis reaction was performed using cell lysis buffer, followed by sonication. Finally, 300ul of cell suspension was incubated with 3ug of antibody (anti-H3K27Ac or normal IgG) overnight at 4°C and then incubated with beads for 4-6 hours to obtain the chromatin and protein complexes. The enriched DNA was tested using specific primers for the promoter region of YBX1 by Quantitative Real-time PCR (QPCR). ChIP primers were showed in **Supplemental Table 1**. Moreover, QPCR products were detected using 1.0% agarose gel electrophoresis containing Ethidium bromide (EtBr). The nucleic acid band were imaged using Chemiluminescence instrument (BioRad, USA).

### Dual Luciferase Reporter Assay

The wild-type and mutant-type of the promoter region of YBX1 were co-transfected into shCtrl or shFOXD3-AS1 stable HOEN1 and SUNE1 cells. Every group was co-transfected with internal reference reporter gene Renilla plasmid. The luciferase activity was detected by Dual-Luciferase Reporter Assay Kit (Promega, USA) and following the manufacturer’s guidance. Relative firefly luciferase activity of each group was normalized to the Renilla luciferase activity.

### Tumor Growth and Lung Metastasis Models *In Vivo*

For tumorigenesis model *in vivo*, 12 BALB/c nude mice (4-5 weeks old) were purchased from Charles River Laboratories (Beijing, China). The stable shCtrl or shFOXD3-AS1 SUNE1 cells (1×10^6^) were injected into the flanks of mice, with tumor volume measured every five days. After 30 days growth, tumors of the mice were removed and weighed.

For lung metastasis models, 10 BALB/c nude mice (4-5 weeks old) were used. The stable shCtrl or shFOXD3-AS1 SUNE1 cells (1×10^6^) were injected into the caudal vein of mice. After 60 days feeding, the mice were dissected for their lungs for study. The animal experiments were approved by the Institutional Animal Care and Use Committee, Xiangya Hospital, Central South University.

### *In Situ* Hybridization Assays

The expression of lncRNA FOXD3-AS1 of implant tumor *in vivo* was evaluated with the ISH Kit (Boster Biological Technology; China). The ISH probes targeting lncRNA FOXD3-AS1 were obtained from Boster and ISH assays were performed according to the manufacturer’s procedure.

### Immunohistochemistry Assays

The expression of YBX1 of implant tumor *in vivo* was examined by immunohistochemistry assays. The slices were baked, dewaxed, and programmed hydration, then incubated with anti-YBX1 antibodies (1:200, CST, USA) at 4°C overnight, and next day were incubated with biotin-labeled corresponding secondary antibody. After the slices were stained with DAB (Agilent Technologies, USA) and counterstained with hematoxylin, the images were captured using NIKON ECLIPSE confocal microscope (Niko, Japan).

### Hematoxylin-Eosin Staining Assays

The sections of xenograft tumors were dewaxed, stained with hematoxylin and eosin, dehydrated, and then sealed with neutral gum (Bioworld, USA) to observe the specimens under a microscope.

### Statistical Analyses

Statistical analysis of the data was performed with SPSS 24.0 software (SPSS, Chicago, IL, USA); P < 0.05 was statistically significant. The comparison of the expression levels of lncRNA FOXD3-AS in cell lines or datasets (GSE61218 and GSE126683) and the data of functional studies between two groups were analyzed by t-test.

## Results

### LncRNA FOXD3-AS1 Is Overexpressed in NPC Tissues and Cell Lines

**Figure 1:**
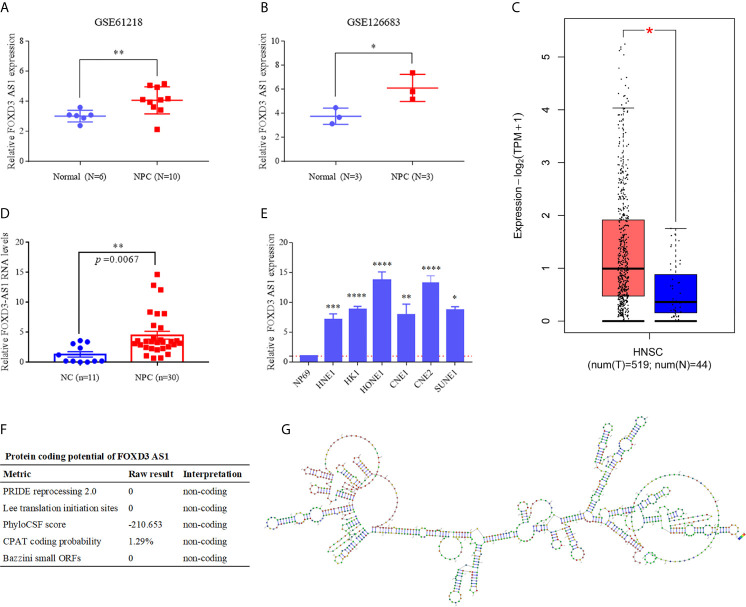
LncRNA FOXD3-AS1 is overexpressed in NPC and has no protein coding function **(A)** The relative expression of LncRNA FOXD3-AS in NPC (n = 10) and normal tissues (n = 6) based on data from the GSE database (GSE61218). **(B)** The relative expression of LncRNA FOXD3-AS in NPC (n = 3) and normal tissues (n = 3) based on data from the GSE database (GSE126683). **(C)** The relative expression of LncRNA FOXD3-AS1 in Head and neck squamous cell carcinoma in TCGA. **(D)** The relative expression of LncRNA FOXD3-AS1 in NPC tissues and nasopharyngeal epithelium (served as a negative control). **(E)** The relative expression of LncRNA FOXD3-AS1 in NPC cell lines and the human nasopharyngeal carcinoma epithelial cell (NP69) detected by RT-qPCR, normalized to GAPDH. **(F)** LncRNA FOXD3-AS1 is disable of protein coding, predicted by LNCipedia. **(G)** The predicted secondary structure of lncRNA FOXD3-AS1 determined by AnnoLnc. *P < 0.05, **P < 0.01, ***p < 0.001, ****p < 0.0001.

To identified the expression of lncRNA FOXD3-AS1 in NPC, first we compared its expression levels with normal nasopharyngeal tissues through GSE61218 and GSE 126683. In both data sets, the expression of FOXD3-AS1 in NPC tissues was statistically upregulated compared with normal nasopharyngeal epithelium tissues (**Figures 1A, B**, P<0.05). In addition, FOXD3-AS1 was over-expressed in Head and neck squamous cell carcinoma in TCGA (**Figure 1C**). As for the NPC tissues, FOXD3-AS1 was also highly expressed (**Figure 1D**). Then, we studied the expression in six nasopharyngeal carcinoma cell lines(HNE1, HK1, HONE1, CNE1, CNE2 and SUNE1, **Figure 1E**) and an immortalized normal nasopharyngeal epithelial cell line (NP69, **Figure 1E**) through RT-qPCR and found that the expression of FOXD3-AS1 was significantly upregulated in NPC cell lines (**Figure 1E**, P<0.05).

### Protein-Free Translation and Structural Prediction of lncRNA FOXD3-AS1

We predicted that lncRNA FOXD3-AS1 had no ability to encode proteins by the database LNCipedia (https://lncipedia.org/) (**Figure 1F**) and the secondary structure of FOXD3-AS1 (http://annolnc.gao-lab.org/) (**Figure 1G**).

### LncRNA FOXD3-AS1 Facilitated the Malignant Progression of NPC *In Vitro*

**Figure 2:**
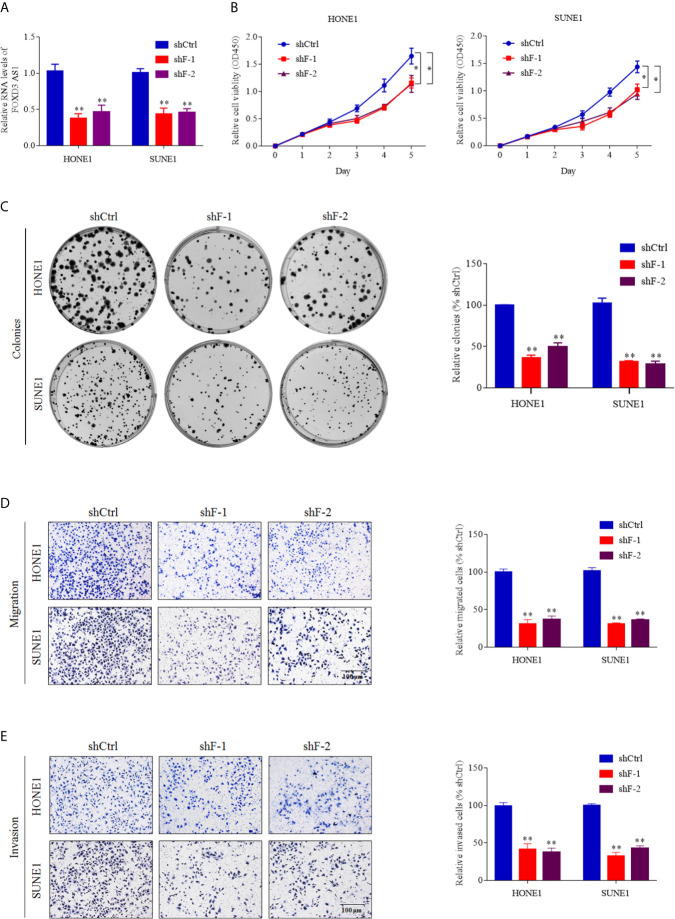
LncRNA FOXD3-AS1 promotes NPC cell proliferation and metastasis *in vitro*. **(A)** Short hairpin RNAs targeting FOXD3-AS1 (shF-1 and shF-2) were adopted to knockdown FOXD3-AS1 in SUNE-1 and HONE-1 cells. The efficiency of knockdown was defined by RT-qPCR. **(B)** Cell proliferation was detected by CCK-8 assays. **(C)** Cell proliferation was detected by colony formation assays. **(D, E)** Representative images (left panel) and statistical analysis (right panel) of transwell migration **(D)** and invasion **(E)** assays in SUNE-1 and HONE-1 cells with or without silenced FOXD3-AS1.Scale bar: 200 μm. *P < 0.05, **P< 0.01.

To elucidate the effect of FOXD3-AS1 in NPC cell lines, we transfected SUNE1 and HONE1 cells with shRNAs of FOXD3-AS1 (shF-1 and shF-2) and shRNA-vector (shCtrl) transiently to silence FOXD3-AS1 (**Figure 2A**). The CCK-8 assays indicated that the NPC cells with downregulated expression of FOXD3-AS1 showed decreased proliferation (**Figure 2B**, P<0.05). The same conclusion was reached in the plate clone formation assays: the number of colonies was lowered when knockdown of FOXD3-AS1 was conducted (**Figure 2C**, P<0.05). When come to the transwell assays, it suggested that the knockdown of FOXD3-AS1 ended up with weakened ability of migration (**Figure 2D**, P<0.05) and invasion (**Figure 2E**, P<0.05).

### LncRNA FOXD3-AS1 Specifically Targeted YBX1 and Regulated the Expression of YBX1

**Figure 3:**
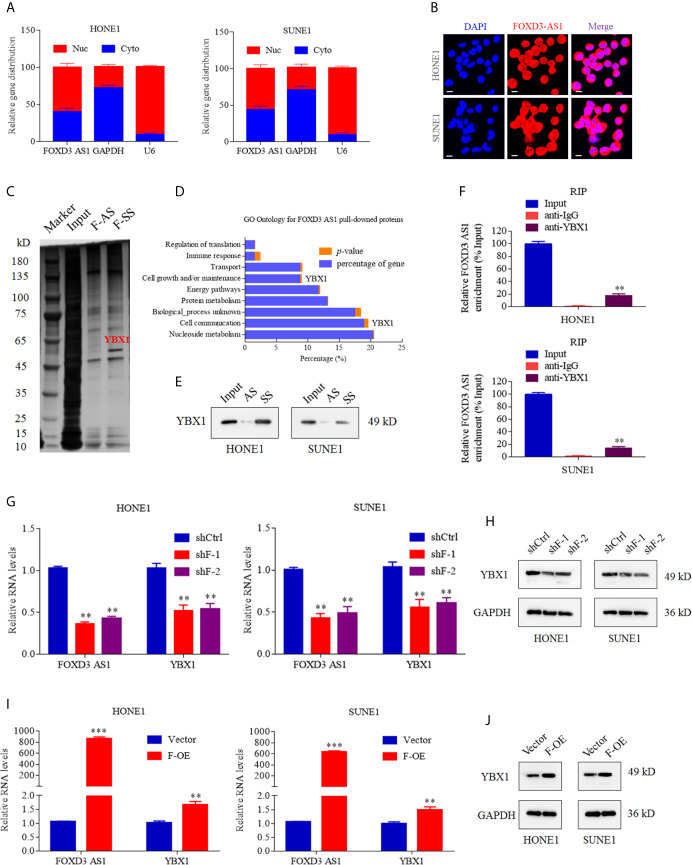
LncRNA FOXD3-AS1 binds to YBX1 and affects the expression of YBX1. **(A)** Relative distribution of FOXD3-AS1 in the nucleus and cytoplasm determined by nucleo-cytoplasmic separation experiments in SUNE1 and HOEN1 cells. Relative expression distribution of GAPDH and U6 served as internal reference of cytoplasm and nucleus, respectively. **(B)** FISH analysis of the enriched level of FOXD3-AS1 in nucleus of the SUNE1 and HOEN1 cells. **(C)** Silver staining images of PAGE gels, in which lncRNA FOXD3-AS1/proteins complexes from RNA pull down assays were separated; Through Liquid chromatography-Mass Spectrometry (LC-MS),YBX1 was defined as the specific protein in pull down complexes by FOXD3-AS1 sense sequence (F-SS),compared with antisense sequence (F-AS). **(D)** GO enrichment analysis was performed according to the pulled down proteins. **(E)** Western bolt verified the combination between YBX1 and FOXD3-AS1 sense sequence (F-SS) compared with antisense sequence (F-AS). **(F)** The RT-qPCR experiments were used to compare the amount of LncFOXD3-AS1 in the YBX1/RNAs complexes obtained from the RNA immunoprecipitation (RIP) in HONE1 (upper) and SUNE1 (lower) cells. **(G)** The knockdown of FOXD3-AS1 expression by Short hairpin RNAs targeting FOXD3-AS1 (shF-1 and shF-2) induced reduction of YBX1 transcription in HONE1 (left) and SUNE1 (right) cells. **(H)** The knockdown of FOXD3-AS1 expression by Short hairpin RNAs targeting FOXD3-AS1 (shF-1 and shF-2) induced reduction of YBX1 translation in HONE1 (left) and SUNE1 (right) cells. **(I)** The over expression of FOXD3-AS1 (F-OE) led to upregulated transcription of YBX1 in HONE1 (left) and SUNE1 (right) cells. **(J)** The over expression of FOXD3-AS1 (F-OE) led to upregulated translation of YBX1 in HONE1 (left) and SUNE1 (right) cells. **P< 0.01, ***p < 0.001.

We identified the subcellular localization of lncRNA FOXD3-AS1 by nucleo-cytoplasmic separation experiment and FISH assay. It was clarified that FOXD3-AS1 existed in both the nucleus and cytoplasm (**Figure 3A**) and the enriched level of FOXD3-AS1 in nucleus of the SUNE1 and HOEN1 cells was detected by FISH assay (**Figure 3B**). In order to seek the target proteins of FOXD3-AS1,we conducted RNA pull down assay, mass spectrometry(MS),RNA RIP assay and western blot. As for RNA pull down assay (**Figure 3C**), the GO analysis of the pulled protein was shown in **Figure 3D** and YBX1 was defined as the specific protein binding with FOXD3-AS1 (**Supplemental Table 2**) after sequencing analysis and western bolt. In western bolt, protein pulled was incubated with anti-YBX1 and we discovered that FOXD3-AS1 sense sequence (F-SS) could bind to YBX1 rather than anti-sense (F-AS) (**Figure 3E**). To further validated that, RNA RIP assay was conducted. RNA combined with Anti-YBX1 was applied with RT-qPCR assay by specific primers of FOXD3-AS1 and it was detected that anti-YBX1 pulls more FOXD3-AS1 than anti-IgG, indicating that FOXD3-AS specifically targeted YBX1 (**Figure 3F**). Then, we tried to explore the relationship of the expression of FOXD3-AS1 and YBX1. When the expression of FOXD3-AS1 was decreased by shRNAs (shF-1 or shF-2), the transcription and translation of YBX1 were also suppressed in NPC cell lines (**Figures 3G, H**). When the expression of FOXD3-AS1 was activated, the transcription and translation of YBX1 were also upregulated (**Figures 3I**, **J**).

### LncRNA FOXD3-AS1 Modulated the Expression of YBX1 by Recruiting H3K27ac

**Figure 4:**
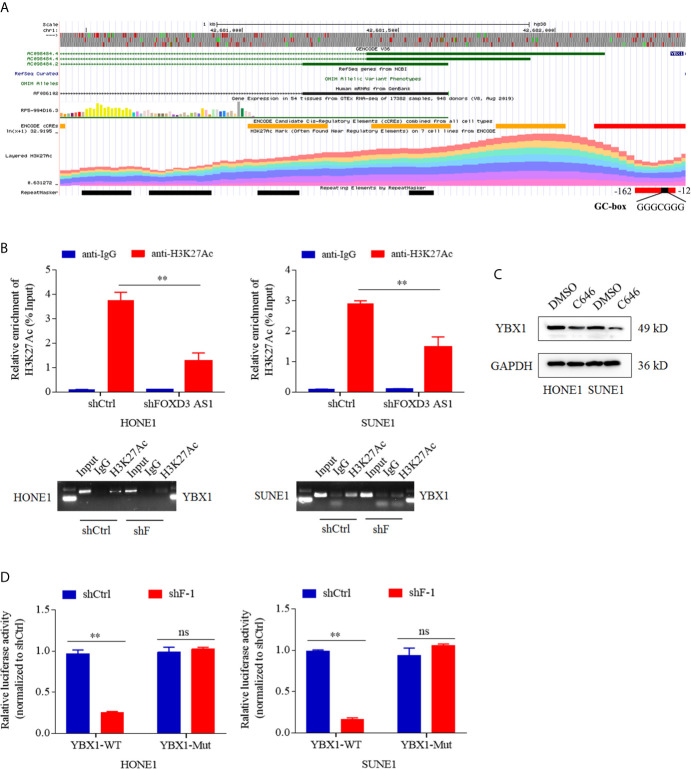
LncRNA FOXD3-AS1 modulated the expression of YBX1 by recruiting H3K27ac. **(A)** Bioinformatics analysis suggested that the promoter of YBX1 had a high enrichment of H3K27ac. **(B)** The relative enrichment of H3K27ac in the promoter of YBX1 was detected by ChIP assays and Quantitative Real-time PCR (QPCR) (upper). Products of QPCR were tested with agarose gel electrophoresis (AGE) assays (lower). **(C)** That deacetylation of H3K27ac suppressed the expression of YBX1 was detected by Western blot. When cells were treated with C646, a histone acetyltransferase inhibitor, the expression of YBX1 was down-regulated, whereas DMSO served as a control. **(D)** That the silence of FOXD3-AS1 suppressed the relative luciferase activity caused by combination of the wild type promoter of YBX1 (YBX1-WT) and H3K27ac was defined by dual luciferase reporter assays in HONE1 cells (left) and SUNE1 cells (right), whereas the luciferase activity induced by the combination of mutated type promoter of YBX1 (YBX1-Mut) and H3K27ac served as a control. NS meant no statistical significance (P<0.05). **P < 0.01.

Why lncRNA FOXD3-AS1 could modulate the expression of YBX1 drew our attention. Bioinformatics analysis showed that the promoter of YBX1 could bind H3K27ac (**Figure 4A**). To validate that, chromatin immunoprecipitation (CHIP)and electrophoresis on agarose gel assays were performed. The specific antibody of H3K27ac was used to screen out the DNA fragments combined with H3K27ac, after which QPCR was conducted with the primer of YBX1 promoter region. The relative enrichment of H3K27ac (%input) in shFOXD3-AS1 group was lower than that in shControl group (**Figure 4B** upper, P<0.05). The gel electrophoresis of the products of QPCR revealed that knockdown of FOXD3-AS1 decreased the enrichment of H3K27ac of YBX1 promoter region (**Figure 4B** lower, P<0.05). Thus, the conclusion was that FOXD3-AS1 could promote the enrichment of H3K27ac in the promoter region of YBX1. In luciferase assays, when decreased FOXD3-AS1, the relative luciferase activity generated by the combination of wild-type promoter of YBX1 and H3K27ac was weakened (**Figure 4D**, P<0.05), prompting that FOXD3-AS1 could promote H3K27ac enrichment in YBX1 promoter region.C646 was an inhibitor of histone acetyltransferase, which could inhibit the acetylation of H3K27. We applied C646 to nasopharyngeal carcinoma cells and used DMSO as a control. Then we found that the expression of YBX1 was lowered when the acetylation of H3K27 was blocked by C646, indicating that H3K27ac could affect the expression of YBX1 (**Figure 4C**). In all, FOXD3-AS1 could regulate the expression of YBX1 by recruiting H3K27ac.

### YBX1 Can Reverse the Proliferation, Migration and Invasion Induced by the Knockdown of lncRNA FOXD3-AS1

**Figure 5:**
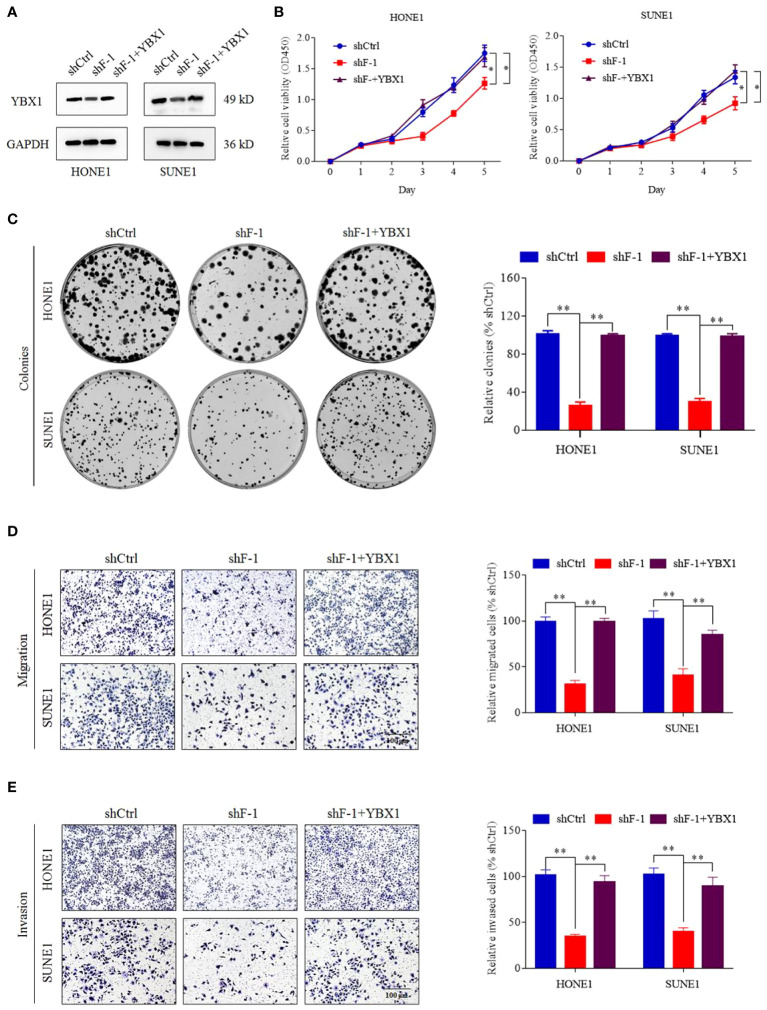
YBX1 reversed the proliferation, migration and invasion induced by knockdown of lncRNA FOXD3-AS1. **(A)** The expression of YBX1 was upregulated by transient transfection in cells silencing FOXD3-AS1 (shF-1), which was detected by western blot. **(B, C)** That YBX1 reversed the proliferation induced by knockdown of lncRNA FOXD3-AS1 was demonstrated by CCK8 assays **(B)** and colony forming assays **(C)** in HONE1 and SUNE1 cells. Colonies of colony forming assays were counted and compared by histogram (**C**, right). **(D, E)** That YBX1 reversed migration **(D)** and invasion **(E)** induced by knockdown of FOXD3-AS1 was validated by transwell assays in HONE1 and SUNE1 cells. Cells of transwell assays were calculated and compared by histograms [**(D, E)**, right]. *P < 0.05, **P < 0.01.

In order to investigate whether FOXD3-AS1 promoted tumor progression by targeting YBX1, we conducted a series of assays. We upregulated the expression of YBX1 in cells with knockdown of FOXD3-AS1 by transient transfection of over-expressed YBX1 plasmids (**Figure 5A**) and observed that the proliferation, migration and invasion of NPC cells were activated through CCK8 (**Figure 5B**), colony formation (**Figure 5C**), migration (**Figure 5D**) and transwell (**Figure 5E**) assays. All results demonstrated that YBX1 could reverse the suppression of tumor progression caused by knockdown of FOXD3-AS1.

### LncRNA FOXD3-AS1 Promoted the Malignant Progression of NPC *In Vivo*

**Figure 6:**
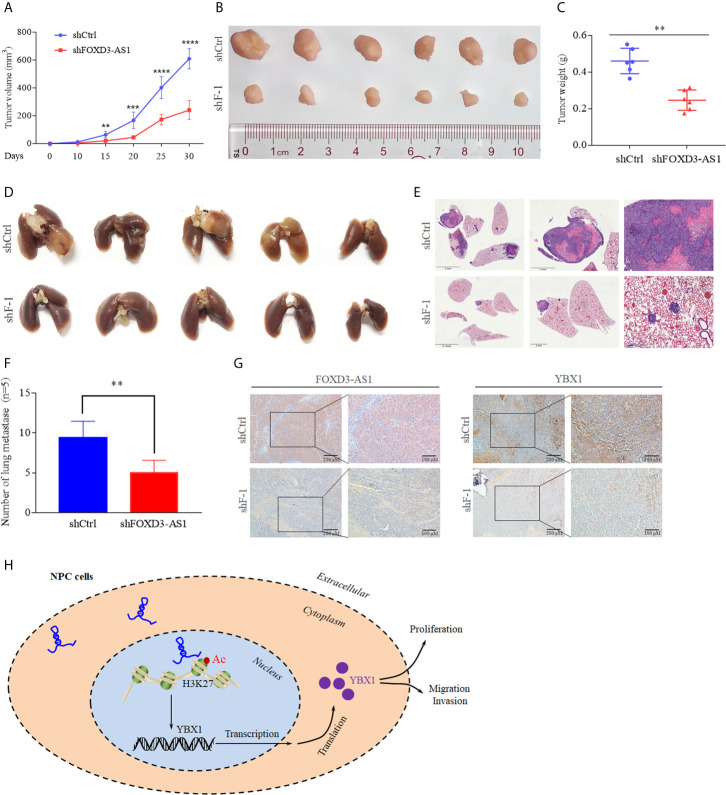
Animal experiments and the mechanism model diagram. **(A, B)** Tumor growth models **(A)** and the tumor volume of mice was reduced after knockdown of FOXD3-AS1 **(B)**. **(C)** Tumor weight was reduced in mice after knockdown of FOXD3-AS1. **(D)** Lung metastasis models of mice. There were less pulmonary metastases in the mice with knockdown of FOXD3-AS1 (lower) than in the mice of the control group (upper). **(E)** Hematoxylin-eosin staining assays demonstrated that knockdown of FOXD3-AS1 induced fewer lung metastases compared with the control group. **(F)** The number of pulmonary metastases of mice was compared between mice with knockdown of FOXD3-AS1 and the control group. **(G)** *In situ* hybridization and immunohistochemistry showed the expression of FOXD3-AS1 (left) and YBX1 (right) in tissue of mice with knockdown of FOXD3-AS1 and the control group. **(H)** Schematic of FOXD3-AS1 mechanism in nasopharyngeal carcinoma. **P < 0.01, ***p < 0.001, ****p < 0.0001.

We constructed tumor growth and metastasis models to confirm the role of lncRNA FOXD3-AS1 in malignant progression of NPC *in vitro*. We injected mice with NPC cells transient transfected with shRNA FOXD3-AS1 and shRNA-vector simultaneously and observed the growth of the xenograft tumors. It was discovered that xenograft tumors with knockdown of FOXD3-AS1 had smaller (**Figures 6A, B**, P<0.05), lighter tumors (**Figure 6C**, P<0.05) and less lung metastasis (**Figures 6D–F**, P<0.05). *In situ* hybridization showed that the expression of FOXD3-AS1 in tumor tissues was decreased after transfecting shRNA FOXD3-AS1 (**Figure 6G**, left). Immunohistochemistry assay indicated that the expression of YBX1 decreased after knockdown of FOXD3-AS1 (**Figure 6G**, right).

## Discussion

More than 100,000 lncRNAs have been identified, but due to the incapability of encoding proteins, most are ignored in the past. However, with a better understanding of the expression and regulation of human gene, people gradually realized the importance of lncRNAs in physiological processes of cells such as chromatin structure remodeling, transcription splicing and editing, mRNA stability, and multidirectional differentiation of stem cells through interaction with DNA, RNA, and proteins (21). Previous studies indicated that some lncRNAs are abnormally expressed in NPC and are involved in the progression of tumor, resistance of chemotherapy and radiation (22–24). LncRNAs can not only inhibit but also promote cancers. Methylation of the promoter region leads to the inactivation of lncRNA MEG3. When lncRNA MEG3 is up-regulated, the proliferation of NPC cells can be inhibited by inducing cell cycle arrest (25). LncRNA LET and LINC0086 also play an important role in NPC suppression (26, 27). With regard to carcinogenesis, lncRNA HOTAIR can activate angiogenesis by stimulating signaling pathway, thus promoting the progression of NPC (28). In our study, we find and verify that abnormal expression of lncRNA FOXD3-AS1 in NPC leads to the proliferation and invasion of NPC by activating the expression of YBX1 by recruiting H3K27ac.

LncRNA FOXD3-AS1 has been identified as high expression in a variety of cancers (29, 30). In cervical cancer, the expression of FOXD3-AS1 is significantly increased and associated with poor differentiation. Down-regulated miR-128-3p and up-regulated LIMK1 can be induced by FOXD3-AS1, which contribute to cancer progression (31). As for osteosarcoma, highly expressed FOXD3-AS1 plays a key role combining miR-296-5p to augment ZCCHC3 (32). When come to hepatocellular carcinoma, the relationship of FOXD3-AS1, miR-335, and RICTOR can explain the mechanism of tumorigenesis and progression (33). In our study, we have found the unusual expression of FOXD3-AS1 in NPC tissues through bioinformatics analysis of GSE61218 and GSE 126683, which is consistent with other datasets (BioProject: PRJNA451367 and GSE64634) in previous studies (18, 34) and demonstrated it in NPC cells and tissues. In addition, FOXD3-AS1 is highly expressed in head and neck squamous cell carcinoma in TCGA database (**Figure 1C**). We further study the relationship between FOXD3-AS1 and NPC progression. In our study, FOXD3-AS1 can promote the proliferation, invasion and metastasis both *in vivo* and *vitro*, which is consistent with previous study (18). In terms of mechanism, past studies focus on micro RNAs as the downstream targets for FOXD3-AS1. For example, the silence of FOXD3-AS1 can inhibit cell growth and apoptosis in NCP cells *via* regulating expression of miR-135a-5p (35). MicroRNA-185-3p is picked as the direct target of FOXD3-AS1 in J Hu et al.’s study (18). However, the networks of gene regulation are very complex. Apart from targeting some micro RNAs, we are interested in whether lncRNA FOXD3-AS1 can influence specific proteins to regulate the development of NPC, which are rarely reported before. Using RNA pull down, MS, RIP assays, western blot, and sequencing analysis, YBX1 is finally detected as a downstream target of FOXD3-AS1. In fact, both YBX1 and MYL6 are top ranked candidates in mass spectrometry analysis and worth studying. However, the functions of YBX1 were outstanding in other tumors (36–38), which caught our attention. When we downregulated the expression of FOXD3-AS1, the expression of YBX1 was also suppressed in a pre-experiment. Thus, we finally identified YBX1 as our target downstream gene. That the expression of YBX1 can be influenced by FOXD3-AS1 is first reported by our research. Through database search, we found that YBX1 was closely related to FOXD3-AS1 in a variety of tumors, which was consistent with our conclusion (**Supplementary Table 3**). YBX1 serves as an oncogene in many cancers, including nasopharyngeal cancer (39–41). Our study also reported similar results. When we up-regulated the expression of YBX1, we reversed proliferation and invasion caused by silencing FOXD3-AS1 in NPC cells (**Figure 5**), suggesting the carcinogenic effect of YBX1. There are many lncRNAs existed that can interact with YBX1. Zhang E et al. have reported that lncRNA HOXC-AS1 can promote the development of gastric cancer combining YBX1 (42). LncRNA DSCAM-AS1 can interacts with YBX1 to facilitate cancer progression (43). In our study, lnc RNA FOXD3-AS1 can combine with YBX1 directly and lead to progression of NPC. All suggested that YBX1 may be a target gene of FOXD3-AS1 affecting tumor development.

What’s more, we confirm that FOXD3-AS1 can increase the expression of YBX1 by enriching H3K27ac in the promoter region of YBX1 (**Figure 4**). H3K27, a member of histones, can make up nucleosomes combine with DNA. Histone post-translational modification is an epigenetic mechanism that regulates gene expression. More precisely, histone acetylation is usually associated with open chromatin structure. Thus, chromatin can be in contact with transcription factors and can significantly increase the level of gene expression (44). Acetylation of histone 3 at lysine 27 (H3K27ac) is related to cellular regulatory processes and can lead to oncogenesis. In esophageal squamous cell carcinoma, H3K27ac can induce high expression of CCAT1 by enriching in the promoter region of CCAT1, and the silence of CCAT1 can inhibit cell proliferation and metastasis *in vivo* and *vitro* (45). High expression of HDAC1 and HDAC2 in hepatocellular carcinoma cells can reduce the acetylation level of H3K27 in the enhancer region of FBP1, and the use of HDAC1 and HDAC2 inhibitors can restore the acetylation of H3K27 in the enhancer region of FBP1, thereby inhibiting glucose metabolism and the growth of liver cancer cells (46). Herein, after deacetylation of the NPC cells, the expression of YBX1 was reduced in our research, which shows that YBX1 is regulated by H3K27ac. In all, we use a mechanism diagram to illustrate process above briefly (**Figure 6H**).

## Conclusion

Finally, our study proved that LncRNA FOXD3-AS1 could promote proliferation and metastasis of NPC by enhancing the transcription of YBX1 by H3K27Ac modification, which may provide new insights into mechanism of the progression of NPC.

## Data Availability Statement

The original contributions presented in the study are included in the article/**Supplementary Material**. Further inquiries can be directed to the corresponding author.

## Ethics Statement

The animal study was reviewed and approved by The Ethics and Research Committees and Department of Oncology, Xiangya Hospital, Central South University, Changsha, China.

## Author Contributions

HY: Experimental studies, data acquisition, and preparation. YP and JZ: Statistical analysis. LJ: Data analysis and editing. XZ: Concepts, review, and supervision. All authors contributed to the article and approved the submitted version.

## Conflict of Interest

The authors declare that the research was conducted in the absence of any commercial or financial relationships that could be construed as a potential conflict of interest.

## Publisher’s Note

All claims expressed in this article are solely those of the authors and do not necessarily represent those of their affiliated organizations, or those of the publisher, the editors and the reviewers. Any product that may be evaluated in this article, or claim that may be made by its manufacturer, is not guaranteed or endorsed by the publisher.

## Abbreviations

LncRNAs, long noncoding RNAs; shRNAs, short hairpin RNAs; NPC, nasopharyngeal carcinoma; RT-qPCR, real-time reverse transcription quantitative; shRNAs, short hairpin RNAs; MS, mass spectrometry; RIP, RNA Immunoprecipitation; TNM, The tumor, node, metastasis; CHIP, chromatin immunoprecipitation; OS, overall survival; GEO, Gene Expression Omnibus; SAM, significant analysis of microarray; RPMI, Roswell Park Memorial Institute; FBS, fetal bovine serum; KSFM, Keratinocyte serum-free medium; QPCR, Quantitative Real-time PCR; FISH, Fluorescence *in situ* hybridization.

## References

[1] Tsao SW Tsang CM Lo KW. Epstein-Barr Virus Infection and Nasopharyngeal Carcinoma. Philos Trans R Soc Lond B Biol Sci (2017) 372(1732):20160270. doi: 10.1098/rstb.2016.0270 28893937 PMC5597737

[2] Siegel RL Miller KD Jemal A. Cancer Statistics, 2019. CA Cancer J Clin (2019) 69(1):7–34. doi: 10.3322/caac.21551 30620402

[3] Zhang B Mo Z Du W Wang Y Liu L Wei Y. Intensity-Modulated Radiation Therapy Versus 2D-RT or 3D-CRT for the Treatment of Nasopharyngeal Carcinoma: A Systematic Review and Meta-Analysis. Oral Oncol (2015) 51(11):1041–6. doi: 10.1016/j.oraloncology.2015.08.005 26296274

[4] Zhu J Duan B Shi H Li Y Ai P Tian J. Comparison of GP and TPF Induction Chemotherapy for Locally Advanced Nasopharyngeal Carcinoma. Oral Oncol (2019) 97:37–43. doi: 10.1016/j.oraloncology.2019.08.001 31421469

[5] Kong L Hu C Niu X Zhang Y Guo Y Tham IW. Neoadjuvant Chemotherapy Followed by Concurrent Chemoradiation for Locoregionally Advanced Nasopharyngeal Carcinoma: Interim Results From 2 Prospective Phase 2 Clinical Trials. Cancer (2013) 119(23):4111–8. doi: 10.1002/cncr.28324 24037893

[6] Qiu WZ Huang PY Shi JL Xia HQ Zhao C Cao KJ. Neoadjuvant Chemotherapy Plus Intensity-Modulated Radiotherapy Versus Concurrent Chemoradiotherapy Plus Adjuvant Chemotherapy for the Treatment of Locoregionally Advanced Nasopharyngeal Carcinoma: A Retrospective Controlled Study. Chin J Cancer (2016) 35:2. doi: 10.1186/s40880-015-0076-9 26739148 PMC4704429

[7] Tang LQ Li CF Li J Chen WH Chen QY Yuan LX. Establishment and Validation of Prognostic Nomograms for Endemic Nasopharyngeal Carcinoma. J Natl Cancer Inst (2015) 108(1):djv291. doi: 10.1093/jnci/djv291 26467665

[8] Li QJ Mao YP Guo R Huang CL Fang XL Ma J. A Nomogram Based on Serum Biomarkers and Clinical Characteristics to Predict Survival in Patients With Non-Metastatic Nasopharyngeal Carcinoma. Front Oncol (2020) 10:594363. doi: 10.3389/fonc.2020.594363 33363024 PMC7758498

[9] Peng H Zhang J Zhang PP Chen L Tang LL Yang XJ. ARNTL Hypermethylation Promotes Tumorigenesis and Inhibits Cisplatin Sensitivity by Activating CDK5 Transcription in Nasopharyngeal Carcinoma. J Exp Clin Cancer Res (2019) 38(1):11. doi: 10.1186/s13046-018-0997-7 30621723 PMC6325889

[10] Wang D Zeng Z Zhang S Xiong F He B Wu Y. Epstein-Barr Virus-Encoded miR-BART6-3p Inhibits Cancer Cell Proliferation Through the LOC553103-STMN1 Axis. FASEB J (2020) 34(6):8012–27. doi: 10.1096/fj.202000039RR 32306460

[11] Wang Y Chen W Lian J Zhang H Yu B Zhang M. The LncRNA PVT1 Regulates Nasopharyngeal Carcinoma Cell Proliferation *Via* Activating the KAT2A Acetyltransferase and Stabilizing HIF-1α. Cell Death Differ (2020) 27(2):695–710. doi: 10.1038/s41418-019-0381-y 31320749 PMC7206084

[12] Zhou L Liu R Liang X Zhang S Bi W Yang M. Lncrna RP11-624L4.1 is Associated With Unfavorable Prognosis and Promotes Proliferation *Via* the CDK4/6-Cyclin D1-Rb-E2f1 Pathway in NPC. Mol Ther Nucleic Acids (2020) 22:1025–39. doi: 10.1016/j.omtn.2020.10.017 PMC7558227 33078086

[13] Zheng ZQ Li ZX Zhou GQ Lin L Zhang LL Lv JW. Long Noncoding Rna FAM225A Promotes Nasopharyngeal Carcinoma Tumorigenesis and Metastasis by Acting as ceRNA to Sponge miR-590-3p/miR-1275 and Upregulate Itgb3. Cancer Res (2019) 79(18):4612–26. doi: 10.1158/0008-5472.CAN-19-0799 31331909

[14] Liu ZB Tang C Jin X Liu SH Pi W. Increased Expression of Lncrna SNHG12 Predicts a Poor Prognosis of Nasopharyngeal Carcinoma and Regulates Cell Proliferation and Metastasis by Modulating Notch Signal Pathway. Cancer Biomark (2018) 23(4):603–13. doi: 10.3233/CBM-181873 PMC13078585 30452404

[15] Zhao CH Bai XF Hu XH. Knockdown of Lncrna XIST Inhibits Hypoxia-Induced Glycolysis, Migration and Invasion Through Regulating miR-381-3p/NEK5 Axis in Nasopharyngeal Carcinoma. Eur Rev Med Pharmacol Sci (2020) 24(5):2505–17. doi: 10.26355/eurrev_202003_20518 32196601

[16] Chen X Gao J Yu Y Zhao Z Pan Y. Lncrna FOXD3-AS1 Promotes Proliferation, Invasion and Migration of Cutaneous Malignant Melanoma *Via* Regulating Mir-325/MAP3K2. BioMed Pharmacother (2019) 120:109438. doi: 10.1016/j.biopha.2019.109438 31541886

[17] Chen Y Gao H Li Y. Inhibition of LncRNA Foxd3-AS1 Suppresses the Aggressive Biological Behaviors of Thyroid Cancer *Via* Elevating miR-296-5p and Inactivating TGF-β1/Smads Signaling Pathway. Mol Cell Endocrinol (2020) 500:110634. doi: 10.1016/j.mce.2019.110634 31678422

[18] Hu J Pan J Luo Z Duan Q Wang D. Long Non-Coding RNA Foxd3-AS1 Silencing Exerts Tumor Suppressive Effects in Nasopharyngeal Carcinoma by Downregulating FOXD3 Expression *Via* microRNA-185-3p Upregulation. Cancer Gene Ther (2020) 28(6):602–18. doi: 10.1038/s41417-020-00242-z 33204001

[19] Volders PJ Anckaert J Verheggen K Nuytens J Martens L Mestdagh P. Lncipedia 5: Towards a Reference Set of Human Long Non-Coding Rnas. Nucleic Acids Res (2019) 47(D1):D135–9. doi: 10.1093/nar/gky1031 PMC6323963 30371849

[20] Ke L Yang DC Wang Y Ding Y Gao G. AnnoLnc2: The One-Stop Portal to Systematically Annotate Novel lncRNAs for Human and Mouse. Nucleic Acids Res (2020) 48(W1):W230–8. doi: 10.1093/nar/gkaa368 PMC7319567 32406920

[21] Ulitsky I Bartel DP. lincRNAs: Genomics, Evolution, and Mechanisms. Cell (2013) 154(1):26–46. doi: 10.1016/j.cell.2013.06.020 23827673 PMC3924787

[22] Li L Gu M You B Shi S Shan Y Bao L. Long Non-Coding RNA ROR Promotes Proliferation, Migration and Chemoresistance of Nasopharyngeal Carcinoma. Cancer Sci (2016) 107(9):1215–22. doi: 10.1111/cas.12989 PMC5021023 27311700

[23] Cui Z Pu T Zhang Y Wang J Zhao Y. Long non-Coding RNA LINC00346 Contributes to Cisplatin Resistance in Nasopharyngeal Carcinoma by Repressing Mir-342-5p. Open Biol (2020) 10(5):190286. doi: 10.1098/rsob.190286 32397872 PMC7276527

[24] Guo Z Wang YH Xu H Yuan CS Zhou HH Huang WH. LncRNA linc00312 Suppresses Radiotherapy Resistance by Targeting DNA-PKcs and Impairing DNA Damage Repair in Nasopharyngeal Carcinoma. Cell Death Dis (2021) 12(1):69. doi: 10.1038/s41419-020-03302-2 33431817 PMC7801696

[25] Chak WP Lung RW Tong JH Chan SY Lun SW Tsao SW. Downregulation of Long Non-Coding RNA MEG3 in Nasopharyngeal Carcinoma. Mol Carcinog (2017) 56(3):1041–54. doi: 10.1002/mc.22569 27597634

[26] Sun Q Liu H Li L Zhang S Liu K Liu Y. Long Noncoding RNA-LET, Which Is Repressed by EZH2, Inhibits Cell Proliferation and Induces Apoptosis of Nasopharyngeal Carcinoma Cell. Med Oncol (2015) 32(9):226. doi: 10.1007/s12032-015-0673-0 26243049

[27] Guo J Ma J Zhao G Li G Fu Y Luo Y. Long Noncoding Rna LINC0086 Functions as a Tumor Suppressor in Nasopharyngeal Carcinoma by Targeting Mir-214. Oncol Res (2017) 25(7):1189–97. doi: 10.3727/096504017X14865126670075 PMC7841018 28245169

[28] Fu WM Lu YF Hu BG Liang WC Zhu X Yang HD. Long Noncoding RNA Hotair Mediated Angiogenesis in Nasopharyngeal Carcinoma by Direct and Indirect Signaling Pathways. Oncotarget (2016) 7(4):4712–23. doi: 10.18632/oncotarget.6731 PMC4826237 26717040

[29] Zeng Z Zhao G Zhu H Nie L He L Liu J. Lncrna FOXD3-AS1 Promoted Chemo-Resistance of NSCLC Cells *Via* Directly Acting on miR-127-3p/MDM2 Axis. Cancer Cell Int (2020) 20:350. doi: 10.1186/s12935-020-01402-9 32742197 PMC7388492

[30] Wu Q Shi M Meng W Wang Y Hui P Ma J. Long Noncoding RNA Foxd3-AS1 Promotes Colon Adenocarcinoma Progression and Functions as a Competing Endogenous RNA to Regulate SIRT1 by Sponging Mir-135a-5p. J Cell Physiol (2019) 234(12):21889–902. doi: 10.1002/jcp.28752 31058315

[31] Yang X Du H Bian W Li Q Sun H. Foxd3−As1/miR−128−3p/LIMK1 Axis Regulates Cervical Cancer Progression. Oncol Rep (2021) 45(5):62. doi: 10.3892/or.2021.8013 33760158 PMC8020211

[32] Wang L. ELF1-Activated FOXD3-AS1 Promotes the Migration, Invasion and EMT of Osteosarcoma Cells *Via* Sponging miR-296-5p to Upregulate ZCCHC3. J Bone Oncol (2020) 26:100335. doi: 10.1016/j.jbo.2020.100335 33204608 PMC7653078

[33] Liu C Zhang M Zhao J Zhu X Zhu L Yan M. Lncrna FOXD3-AS1 Mediates AKT Pathway to Promote Growth and Invasion in Hepatocellular Carcinoma Through Regulating Rictor. Cancer Biother Radiopharm (2020) 35(4):292–300. doi: 10.1089/cbr.2019.3335 32191537

[34] Xu YZ Chen FF Zhang Y Liang H Li XJ He C. Identification of Potential Long Noncoding RNA Associated With Nasopharyngeal Carcinoma Using Deep Sequencing. J Int Med Res (2019) 47(7):3271–81. doi: 10.1177/0300060519845973 PMC6683893 31122165

[35] Zhang E Li C Xiang Y. Lncrna FOXD3-AS1/miR-135a-5p Function in Nasopharyngeal Carcinoma Cells. Open Med (Wars) (2020) 15(1):1193–201. doi: 10.1515/med-2020-0177 PMC7718651 33336076

[36] Su H Fan G Huang J Qiu X. YBX1 Regulated by Runx3-miR-148a-3p Axis Facilitates Non-Small-Cell Lung Cancer Progression. Cell Signal (2021) 85:110049. doi: 10.1016/j.cellsig.2021.110049 34082012

[37] Song S He X Wang J Song H Wang Y Liu Y. A Novel Long Noncoding RNA, Tmem92-AS1, Promotes Gastric Cancer Progression by Binding to YBX1 to Mediate CCL5. Mol Oncol (2021) 15(4):1256–73. doi: 10.1002/1878-0261.12863 PMC8024739 33247987

[38] Wang M Dai M Wang D Tang T Xiong F Xiang B. The Long Noncoding RNA AATBC Promotes Breast Cancer Migration and Invasion by Interacting With YBX1 and Activating the YAP1/Hippo Signaling Pathway. Cancer Lett (2021) 512:60–72. doi: 10.1016/j.canlet.2021.04.025 33951538

[39] Cui Y Li F Xie Q Zhao S Guo T Guo P. YBX1 Mediates Autophagy by Targeting P110β and Decreasing the Sensitivity to Cisplatin in NSCLC. Cell Death Dis (2020) 11(6):476. doi: 10.1038/s41419-020-2555-4 32561752 PMC7305216

[40] Lim JP Shyamasundar S Gunaratne J Scully OJ Matsumoto K Bay BH. YBX1 Gene Silencing Inhibits Migratory and Invasive Potential *Via* CORO1C in Breast Cancer *In Vitro* . BMC Cancer (2017) 17(1):201. doi: 10.1186/s12885-017-3187-7 28302118 PMC5356414

[41] Zhou LL Ni J Feng WT Yao R Yue S Zhu YN. High YBX1 Expression Indicates Poor Prognosis and Promotes Cell Migration and Invasion in Nasopharyngeal Carcinoma. Exp Cell Res (2017) 361(1):126–34. doi: 10.1016/j.yexcr.2017.10.009 29024700

[42] Zhang E He X Zhang C Su J Lu X Si X. A Novel Long Noncoding RNA Hoxc-AS3 Mediates Tumorigenesis of Gastric Cancer by Binding to YBX1. Genome Biol (2018) 19(1):154. doi: 10.1186/s13059-018-1523-0 30286788 PMC6172843

[43] Zhang Y Huang YX Wang DL Yang B Yan HY Lin LH. Lncrna DSCAM-AS1 Interacts With YBX1 to Promote Cancer Progression by Forming a Positive Feedback Loop That Activates FOXA1 Transcription Network. Theranostics (2020) 10(23):10823–37. doi: 10.7150/thno.47830 PMC7482804 32929382

[44] Roth SY Denu JM Allis CD. Histone Acetyltransferases. Annu Rev Biochem (2001) 70:81–120. doi: 10.1146/annurev.biochem.70.1.81 11395403

[45] Zhang E Han L Yin D He X Hong L Si X. H3K27 Acetylation Activated-Long Non-Coding RNA CCAT1 Affects Cell Proliferation and Migration by Regulating SPRY4 and HOXB13 Expression in Esophageal Squamous Cell Carcinoma. Nucleic Acids Res (2017) 45(6):3086–101. doi: 10.1093/nar/gkw1247 PMC5389582 27956498

[46] Yang J Jin X Yan Y Shao Y Pan Y Roberts LR. Inhibiting Histone Deacetylases Suppresses Glucose Metabolism and Hepatocellular Carcinoma Growth by Restoring FBP1 Expression. Sci Rep (2017) 7:43864. doi: 10.1038/srep43864 28262837 PMC5338333

