# Corrigendum: LncRNA FOXD3-AS1 promotes the malignant progression of nasopharyngeal carcinoma through enhancing the transcription of YBX1 by H3K27Ac modification

**DOI:** 10.3389/fonc.2024.1436609

**Published:** 2024-06-11

**Authors:** Huiyun Yang, Yuliang Pan, Jun Zhang, Long Jin, Xi Zhang

**Affiliations:** ^1^ Department of Oncology, Xiangya Hospital, Central South University, Changsha, China; ^2^ Department of Oncology, Third Xiangya Hospital, Central South University, Changsha, China

**Keywords:** FOXD3-AS1, nasopharyngeal carcinoma, YBX1, H3K27ac, lnc RNA

In the published article, there was an error in **Figure 5** due to incorrect image editing in **Figure 5D** as published. The corrected **Figure 5** appear below.

**Figure 5:**
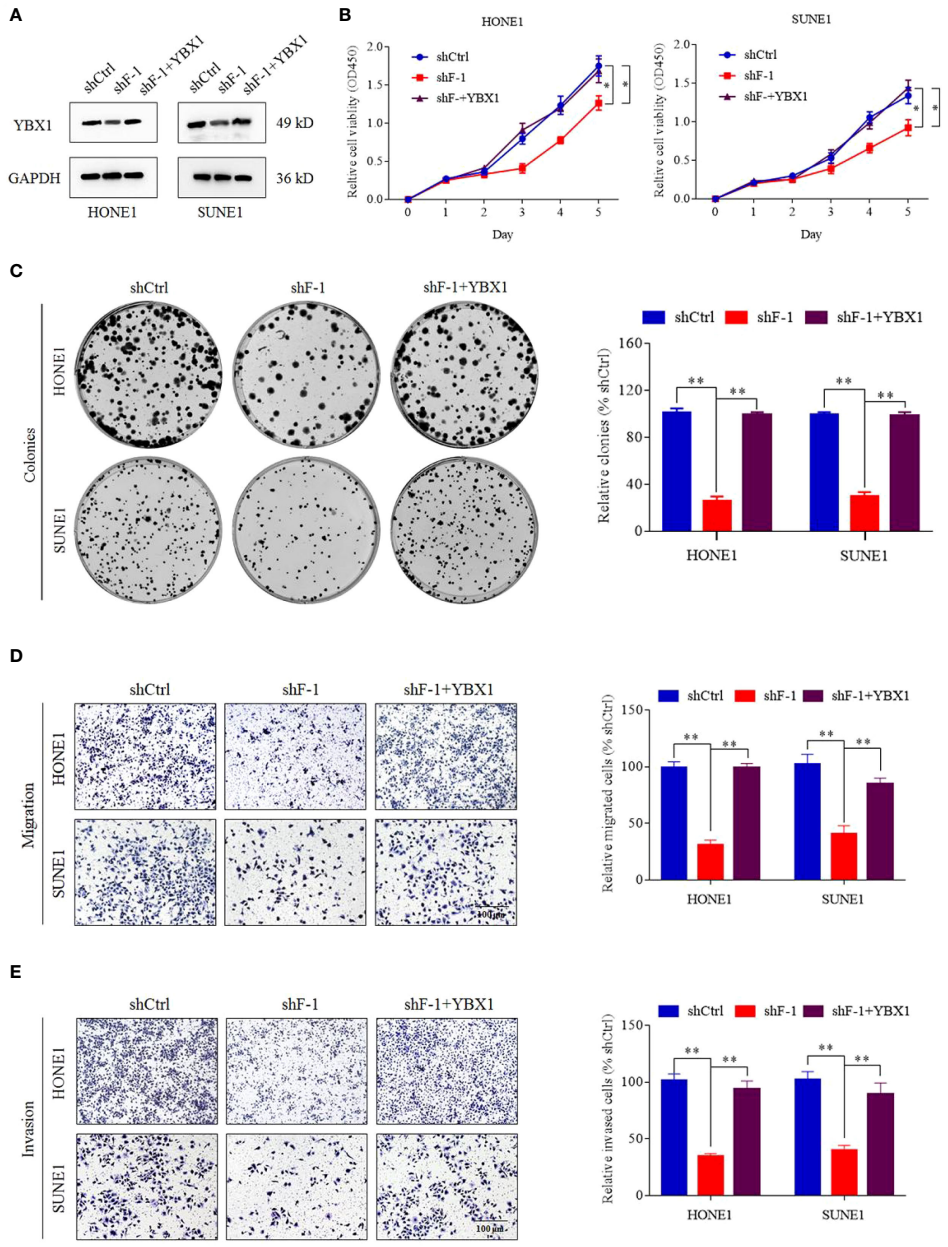
YBX1 reversed the proliferation, migration and invasion induced by knockdown of lncRNA FOXD3-AS1. **(A)** The expression of YBX1 was upregulated by transient transfection in cells silencing FOXD3-AS1 (shF-1), which was detected by western blot. **(B, C)** That YBX1 reversed the proliferation induced by knockdown of lncRNA FOXD3-AS1 was demonstrated by CCK8 assays **(B)** and colony forming assays **(C)** in HONE1 and SUNE1 cells. Colonies of colony forming assays were counted and compared by histogram (**C**, right). **(D, E)** That YBX1 reversed migration **(D)** and invasion **(E)** induced by knockdown of FOXD3-AS1 was validated by transwell assays in HONE1 and SUNE1 cells. Cells of transwell assays were calculated and compared by histograms [**(D, E)**, right]. *P < 0.05, **P < 0.01.

The authors apologize for this error and state that this does not change the scientific conclusions of the article in any way. The original article has been updated.

