# Supplementary material for: LncRNA FOXD3-AS1 Promotes the Malignant Progression of Nasopharyngeal Carcinoma Through Enhancing the Transcription of YBX1 by H3K27Ac Modification
Source: Front Oncol. 2021 Jul 29;11:715635. doi: 10.3389/fonc.2021.715635 (PMC8359730; doi:10.3389/fonc.2021.715635)
Supplement: Supplementary file 1 [file Table_1.docx]

**Supplementary Table 1. Primers for vector construction, qPCR, ChIP-qPCR and shRNAs**

| **Name** | **Forward Primer (5'-3')** |
| --- | --- |
| **Primers for qRT-PCR from cell lines** | |
| FOXD3 AS1 F | GGAGTGCAAGGCCGCCTAGT |
| FOXD3 AS1 R | CTCGGAACTCCTCCCCTCGT |
| YBX1 F | GCAGGAGAACAAGGTAGACCAG |
| YBX1 R | CTTCATTGCCGTCCTCTCTAGG |
| GAPDH F | AACGGATTTGGTCGTATTGG |
| GAPDH R | TTGATTTTGGAGGGATCTCG |
| U6 F | CTCGCTTCGGCAGCACA |
| U6 R | TCTTGTCCTCGCCTTGTCTT |
| **shRNA sequences** | |
| shFOXD3 AS1-1 | GGAAATAATTAGTGAAATA |
| shFOXD3 AS1-2 | GGAGGAGTTCCGAGAGGAA |
| **Primers for FOXD3 AS1 sense and antisense** | |
| Sense F | CGCGGATCCAACAAAGGGACGAGAGACGCG |
| Sense R | CCGCTCGAGGGTGTGTCTAGGCCAAGGAAA |
| Anti-sense F | CGCGGATCCATTGAATTATTTTTGGTGTGT |
| Anti-sense R | CCGCTCGAGAACAAAGGGACGAGAGACGCG |
| **Primers for plasmid constructs** | |
| YBX1 F | CGCGGATCCATGAGCAGCGAGGCCGAGAC |
| YBX1 R | CCGCTCGAGTTACTCAGCCCCGCCCTGCT |
| YBX1 WT F | CGGACGCGTACTGAATTAGCCGCCAAAGG |
| YBX1 WT R | CCGCTCGAGGGGATAAGCCCTACGAGCGA |
| YBX1 Mut F | CGGACGCGTACTGAATTAGCCGCCAAAGG |
| YBX1 Mut R | CCGCTCGAGGGGATAAGCCCTACGAGCGATAGGCGTTGT |
| **Primers for RIP** | |
| FOXD3 AS1-RIP F | GCATGCTGGGATCTCCGTT |
| FOXD3 AS1-RIP R | TGCATTGGCCACACATCCT |
| **Primers for ChIP** | |
| YBX1-1 F | GATGACAGTCCTTTCTCTACG |
| YBX1-1 R | TGTTGCTTCAGTGTATCAGAT |
| YBX1-2 F | TCACAGGCCTAGACGGCATT |
| YBX1-2 R | TTTAGAGTCATAAGTAGGGA |
