# Supplementary material for: LncRNA FOXD3-AS1 Promotes the Malignant Progression of Nasopharyngeal Carcinoma Through Enhancing the Transcription of YBX1 by H3K27Ac Modification
Source: Front Oncol. 2021 Jul 29;11:715635. doi: 10.3389/fonc.2021.715635 (PMC8359730; doi:10.3389/fonc.2021.715635)
Supplement: Supplementary file 2 [file Table_2.doc]

**Supplementary Table 2. The top10 proteins of FOXD3 AS1 RNA pull down by mass spectrometry analysis**

| **Protein** | **Score** | |
| --- | --- | --- |
| Myosin Light Chain 6, MYL6 | | 319 |
| **Y-Box Binding Protein 1, YBX1** | | **284** |
| GNAS Complex Locus, GNAS | | 231 |
| Insulin Like Growth Factor 2 MRNA Binding Protein 2, IGF2BP2 | | 214 |
| Major Histocompatibility Complex, Class I, B, HLA-B | | 189 |
| Peroxiredoxin-2, PRDX2 | | 113 |
| Coiled-Coil-Helix-Coiled-Coil-Helix Domain Containing 3, CHCHD3 | | 85 |
| Member Of RAS Oncogene Family, RAP1B | | 67 |
| Vinculin, VCL | | 65 |
| Translocase Of Outer Mitochondrial Membrane 22, TOMM22 | | 62 |
