# Supplementary figures and images for: LncRNA FOXD3-AS1 Promotes the Malignant Progression of Nasopharyngeal Carcinoma Through Enhancing the Transcription of YBX1 by H3K27Ac Modification
Source: Front Oncol. 2021 Jul 29;11:715635. doi: 10.3389/fonc.2021.715635 (PMC8359730; doi:10.3389/fonc.2021.715635)

Supplementary Table 3. Expression of FOXD3-AS1 in various cancers in TCGA database.


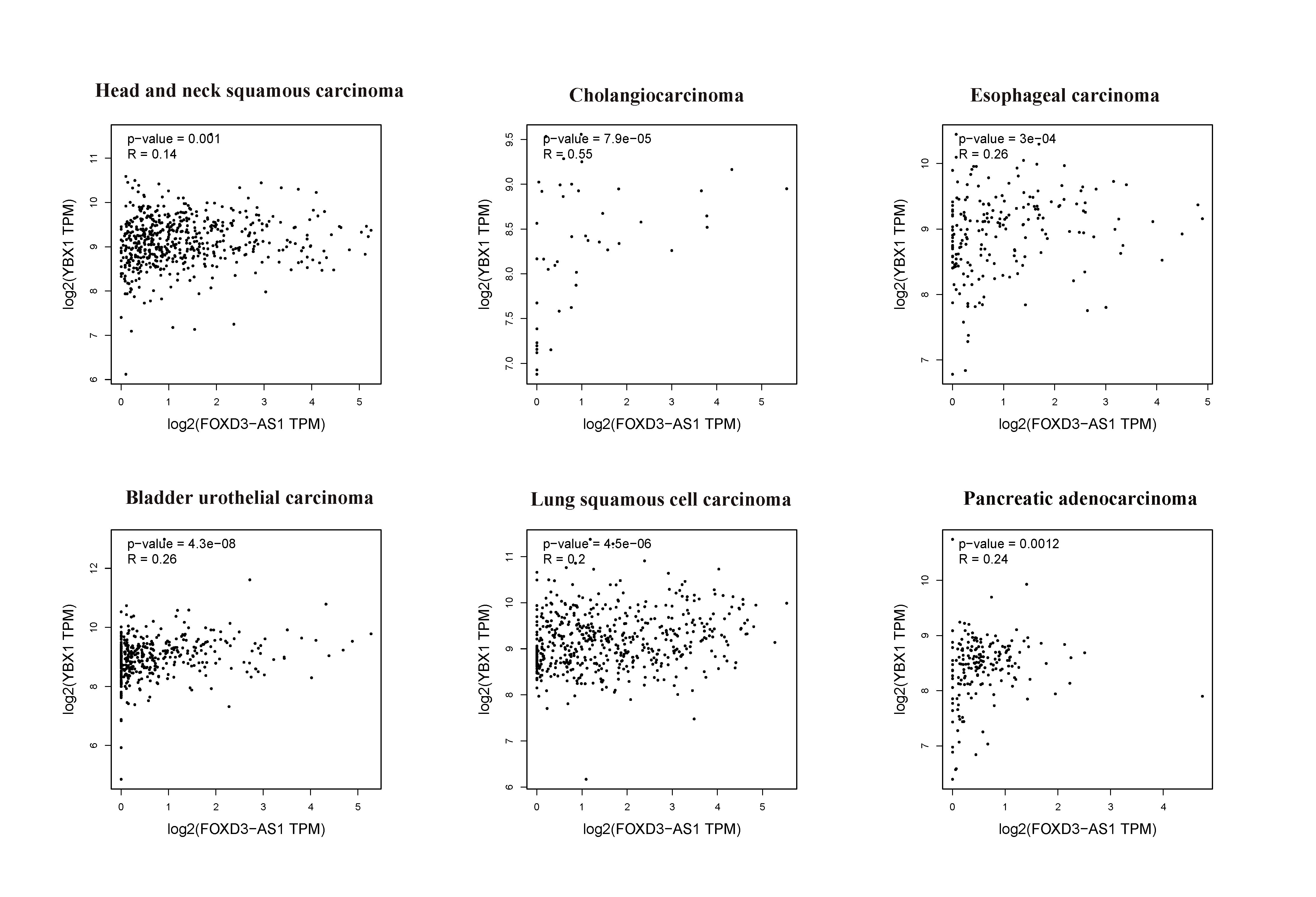

Supplement: Supplementary file 3 [file Table_3.doc]
